# 再生障碍性贫血患者免疫抑制治疗中新型冠状病毒感染临床症状评估——前瞻性NICHE队列研究

**DOI:** 10.3760/cma.j.issn.0253-2727.2023.11.003

**Published:** 2023-11

**Authors:** 婧余 赵, 乐乐 张, 哲湘 匡, 静 徐, 为为 王, 虹 潘, 珍 高, 伟望 李, 力维 方, 振 宋, 均 施

**Affiliations:** 1 中国医学科学院血液病医院（中国医学科学院血液学研究所），实验血液学国家重点实验室，国家血液系统疾病临床医学研究中心，细胞生态海河实验室，天津 300020 State Key Laboratory of Experimental Hematology, National Clinical Research Center for Blood Diseases, Haihe Laboratory of Cell Ecosystem, Institute of Hematology & Blood Diseases Hospital, Chinese Academy of Medical Sciences & Peking Union Medical College, Tianjin 300020, China; 2 天津医学健康研究院，天津 301600 Tianjin Institutes of Health Science, Tianjin 301600, China

**Keywords:** 再生障碍性贫血, 免疫抑制治疗, 新型冠状病毒, Aplastic anemia, Immunosuppressive therapy, COVID-19

## Abstract

**目的:**

调查再生障碍性贫血（AA）患者接受免疫抑制治疗中新型冠状病毒（新冠）感染临床症状及转归。

**方法:**

前瞻性研究2022年12月1日至2023年1月31日期间新冠感染的AA患者人口学特征和临床症状，并分析AA免疫抑制治疗对新冠感染临床特征的影响。

**结果:**

共纳入170例合并新冠感染的AA患者，早期高发症状中发热、头痛或头晕、肌肉或全身酸痛、咽痛这四种表现在第1～2周时明显好转，约25％的患者在2周时持续乏力，咳嗽或咳痰症状在感染1～2周后明显增多。免疫抑制治疗组和非免疫抑制治疗组、免疫抑制治疗持续6个月及以上与短于6个月、曾接受抗淋巴细胞球蛋白（ALG）治疗组和未接受过ALG治疗组的发热天数和最高体温差异均无统计学意义（均*P*>0.05）。新冠感染后1、2周患者症状恢复情况，在是否接受免疫治疗及其治疗时长亚组中差异均无统计学意义（均*P*>0.05）；但曾接受ALG治疗者感染1周的发热比例低于未接受ALG治疗者（*P*＝0.035），感染2周的咽痛比例高于未接受ALG治疗者（*P*＝0.015），其他临床症状差异均无统计学意义（均*P*>0.05）。

**结论:**

AA患者接受免疫抑制治疗中新冠感染后早期症状明显，但大多数可在两周内恢复。

## Abstract (en)

**Objective:**

To investigate the clinical features of coronavirus disease 2019（COVID-19）in patients with aplastic anemia（AA）undergoing immunosuppressive therapy（IST）.

**Methods:**

In this prospective cohort study, we collected the demographic and clinical data of patients with AA and COVID-19 from December 1, 2022, to January 31, 2023. We described the clinical features of COVID-19 among patients with AA and evaluated the effects of IST on the signs and severity of COVID-19.

**Results:**

A total of 170 patients with AA and COVID-19 were included. The common early symptoms, including fever, dizziness or headache, muscle or body aches, and sore throat, disappeared within 1–2 weeks. Approximately 25％ of the patients had persistent fatigue within 2 weeks. Many patients experienced cough after an initial 1–3 days of infection, which lasted for more than 2 weeks. There were no differences in the duration of total fever episodes and maximum body temperature when patients were stratified according to whether or not they underwent IST, by IST duration, or by use of anti-lymphocyte globulin（ALG）（*P*>0.05）. No differences were observed in the occurrence of symptoms in either the early or recovery stages when patients with AA were stratified according to whether or not they underwent IST, or by IST duration（*P*>0.05）. However, patients who received ALG had fewer fever episodes within 1 week after infection（*P*＝0.035）and more sore throat episodes within 2 weeks after infection（*P*＝0.015）. There were no other significant differences in clinical symptoms between patients who did and patients who did not receive ALG（*P*>0.05）.

**Conclusion:**

The majority of patients with AA and COVID-19 recovered within 2 weeks of noticing symptoms when treated with IST

健康社会人群新型冠状病毒（新冠）感染后临床症状轻重程度不一，而弱势的患病人群新冠感染后临床症状评估鲜有报道。新冠感染后机体免疫反应及临床症状可能因合并严重血液系统疾病而有所不同。

再生障碍性贫血（AA）是一类以T淋巴细胞介导骨髓造血损伤的血液系统疾病，表现为一系或多系血细胞减少，部分患者长期处于粒细胞减低/缺乏状态。且患者多接受以环孢素A（CsA）、抗淋巴/胸腺细胞球蛋白（ALG/ATG）为代表的免疫抑制治疗（IST）[1]。AA患者合并新冠感染的风险高，接受IST是否影响感染后临床症状、转归，与健康人/其他血液系统肿瘤患者的临床症状有无差异尚不明确。

基于国家血液病队列研究项目（National Longitudinal Cohort of Hematological Diseases in China，NICHE），我们前瞻性调查170例AA患者合并新冠感染后两周内临床症状及其转归，分析是否接受IST、IST时间及强度对新冠感染临床特征的影响。

## 病例与方法

1. 病例资料：本研究为前瞻、观察性队列研究，患者来源于中国医学科学院血液病医院（中国医学科学院血液学研究所）建立的NICHE队列（伦理批件号：IIT2021008-EC-1）。研究对象纳入标准：①AA诊断及分型依据文献[2]–[3]；②2022年12月1日至2023年1月31日新冠核酸检测或抗原检测阳性，存在典型的新冠感染症状，且具有明确的流行病学史（发病前14 d内与新冠感染者有接触史和聚集性发病）[4]。排除标准：随访期间应答率低或无应答，缺失数据大于20％。

2. 前瞻性随访：AA患者新冠感染首日、3 d、5 d、1周和2周进行随访和资料收集。随访内容主要包括人口学特征、疫苗接种、临床表现、治疗方案及合并用药等。共随访173例患者，3例随访过程中应答率较低，未纳入研究。

3. 治疗方案：①IST组：CsA和（或）ALG药物合并使用促造血药物；②非IST组：单用促造血药物（雄激素、TPO受体激动剂、中药等）或停药观察者。

4. 统计学处理：应用R 4.0.2软件进行数据处理和分析。分类变量采用例数（构成比）、连续变量采用中位数（范围）进行统计学描述。连续变量的组间比较采用Mann-Whitney *U*检验（数据不符合正态分布）。无序分类变量采用Pearson卡方检验或 Fisher 精确概率法进行组间比较，有序分类变量比较采用秩和检验进行组间比较。双侧*P*<0.05为差异有统计学意义。

## 结果

一、一般资料

170例AA患者中位年龄33（10～72）岁，男85例（50.0％），本科及以上文化程度65例（38.3％），身体质量指数（BMI）属于肥胖24例（14.1％），既往或目前吸烟34例（20.0％），合并基础疾病27例（16.0％），未接种新冠疫苗124例（72.9％）。非重型和重型AA分别为123例（72.4％）和47例（27.6％）。170例AA患者中，正在接受IST 151例（88.8％）：IST时间6个月及以上（IST≥6个月）133例（88.1％）；曾接受ALG治疗37例（21.8％）。接受IST组和未接受IST组的人口学特征差异均无统计学意义（均*P*>0.05）（表1）。

**表1:** 170例再生障碍性贫血（AA）患者合并新型冠状病毒感染人口学特征

特征	所有患者（170例）	免疫抑制治疗组（151例）	非免疫抑制治疗组（19例）	统计量	*P*值
性别[例（%）]				Fisher	1.000
男	85（50.0）	76（50.3）	9（47.4）		
女	85（50.0）	75（49.7）	10（52.6）		
年龄[岁，*M*（范围）]	33（10~72）	33（10~72）	33（19~65）	−0.644	0.526
文化程度[例（%）]				−1.620	0.105
高中及以下	74（43.5）	69（45.7）	5（26.3）		
大专	31（18.2）	27（17.9）	4（21.1）		
本科及以上	65（38.3）	55（36.4）	10（52.6）		
BMI[例（%）]				−0.245	0.805
体重过低	10（5.9）	9（6.0）	1（5.3）		
体重正常	85（50.0）	75（49.6）	10（52.6）		
超重	51（30.0）	45（29.8）	6（31.6）		
肥胖	24（14.1）	22（14.6）	2（10.5）		
吸烟史[例（%）]				−0.337	0.736
从不吸烟	136（80.0）	120（79.5）	16（84.2）		
已戒烟	25（14.7）	24（15.9）	1（5.3）		
目前吸烟	9（5.3）	7（4.6）	2（10.5）		
既往病史[例（%）]				5.528	0.355
无	143（84.0）	129（85.5）	14（73.6）		
恶性肿瘤	4（2.4）	3（2.0）	1（5.3）		
肺部感染	4（2.4）	3（2.0）	1（5.3）		
COPD/哮喘	4（2.4）	4（2.6）	0		
内分泌代谢疾病	4（2.4）	4（2.6）	0		
其他	11（6.4）	8（5.3）	3（15.8）		
新冠疫苗接种情况[例（%）]				−1.389	0.165
未接种	124（72.9）	108（71.5）	16（84.2）		
接种1针	7（4.1）	5（3.3）	2（10.5）		
接种2~3针	39（23.0）	38（25.2）	1（5.3）		
诊断亚型[例（%）]				1.759	0.415
TI-NSAA	96（56.5）	84（55.6）	12（63.2）		
TD-NSAA	27（15.9）	23（15.2）	4（21.0）		
SAA/VSAA	47（27.6）	44（29.2）	3（15.8）		

注 COPD：慢性阻塞性肺疾病；TI-NSAA：非输血依赖型AA；TD-NSAA：输血依赖型AA；SAA/VSAA：重型/极重型AA

二、AA患者两周内新冠感染临床症状转归

AA患者新冠感染3 d内临床症状主要包括发热134例（78.8％）、头晕/头痛86例（50.6％）、乏力76例（44.7％）、肌肉或全身酸痛70例（41.2％）、咽痛68例（40.0％）、咳嗽/咳痰57例（33.5％）。发热、头痛/头晕、肌肉或全身酸痛、咽痛这4种表现在第1～2周时明显好转，2周时仍然有该4种表现者比例仅占1％～7％；而乏力改善者比例较小，2周时仍然有约25％的患者感乏力；相反的是，咳嗽/咳痰症状在1周、2周时发生率增高，达50％～70％。味觉/嗅觉减退尽管发生率较低，但2周内基本持续存在（图1）。1例（0.6％）在新冠感染期间进展为危重型病例，并进入重症监护病房接受机械通气治疗；无新冠感染相关死亡病例。

**图1:**
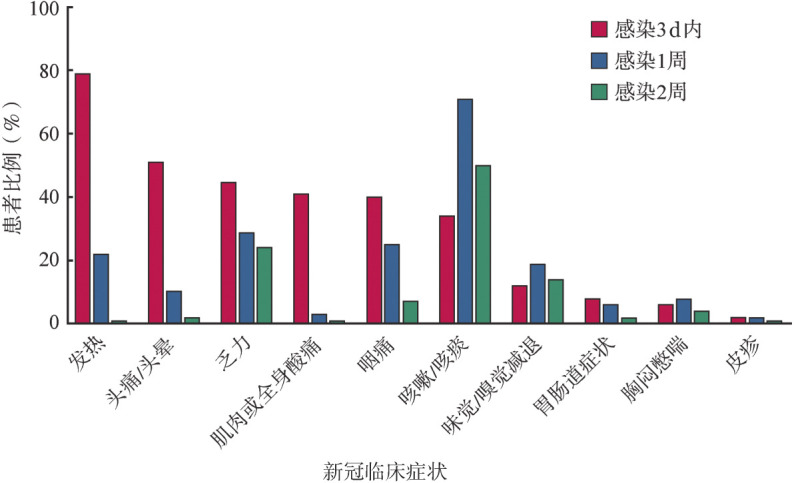
再生障碍性贫血患者新型冠状病毒（新冠）感染2周内临床症状转归

三、AA IST患者新冠感染的特征分析

1. 感染早期（3 d内）临床特征：发热是感染早期主要表现，我们分析了是否接受IST、IST时长、IST强度等亚组之间发热天数和最高体温的差异。IST组和非IST组发热天数和最高体温差异均无统计学意义（*P*＝0.75和*P*＝0.75）；IST≥6个月与IST<6个月者在发热天数和最高体温上差异亦无统计学意义（*P*＝0.49和*P*＝0.93）；曾接受强IST治疗药物ALG的患者和未接受过ALG（无ALG）的患者在发热天数和最高体温上差异均无统计学意义（*P*>0.99和*P*＝0.13）（图2）。早期其他临床症状在IST与非IST、IST≥6个月与IST<6个月、ALG组与无ALG组间比较差异均无统计学意义（均*P*>0.05）（图3）。

**图2:**
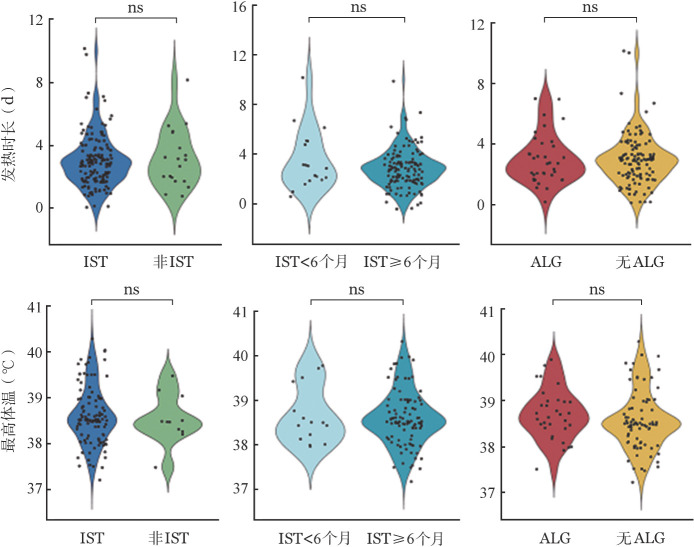
再生障碍性贫血患者免疫抑制治疗（IST）各亚组间发热天数（A）和最高体温（B）比较分析（^ns^*P*>0.05） ALG：抗淋巴细胞球蛋白

**图3:**
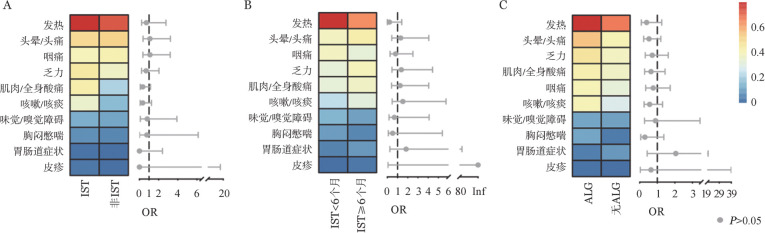
再生障碍性贫血患者免疫抑制治疗（IST）各亚组间早期临床症状发生率比较分析 ALG：抗淋巴细胞球蛋白

2. 感染恢复期（1、2周）临床特征：新冠感染后1、2周患者症状恢复情况，IST组和非IST组感染1周和2周的临床症状发生率差异无统计学意义（*P*>0.05）；IST≥6个月组患者在感染1周和2周的临床症状发生率和IST<6个月组差异均无统计学意义（*P*>0.05）。ALG组感染1周的发热比例低于无ALG组（*P*＝0.035），感染2周的咽痛比例高于无ALG组（*P*＝0.015），其他临床症状发生率在两组间差异均无统计学意义（均*P*>0.05）（图4）。

**图4:**
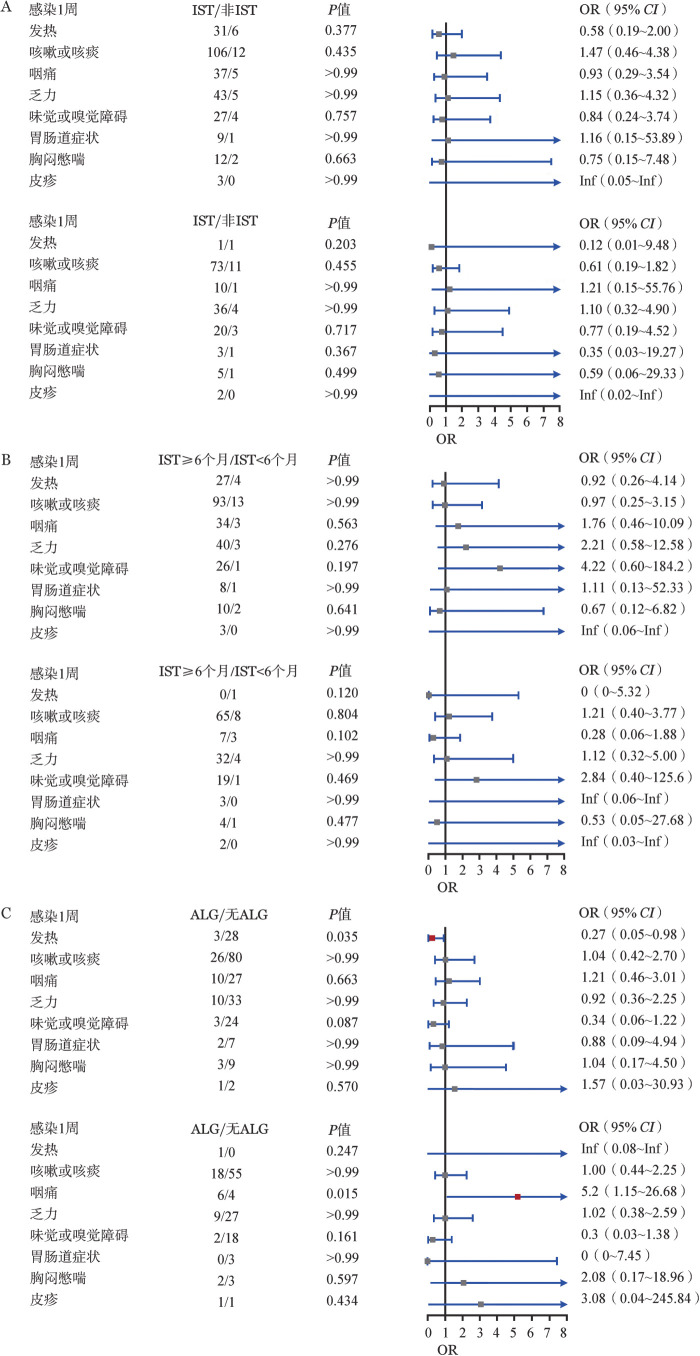
再生障碍性贫血患者免疫抑制治疗（IST）各亚组间恢复期临床症状特征比较分析 ALG：抗淋巴细胞球蛋白

## 讨论

本研究是一项针对AA合并新冠感染患者的前瞻性队列研究。结果显示接受IST AA患者新冠感染后早期症状明显，但大多数可在两周内恢复。将AA患者与感染奥密克戎的430例天津社会人群[5]的临床数据进行比较，我们发现AA患者群体早期临床症状发生率明显高于社会人群，但新冠重症率与社会人群接近。AA是一类T细胞异常活化介导的骨髓造血损伤性疾病[1]，患者感染早期出现发热、乏力、咽痛等症状比例明显高于社会人群，可能与其接受病毒抗原刺激后机体免疫应答异常活化相关，导致早期出现更明显的病理反应；AA患者与社会人群重症率相近，提示骨髓造血衰竭所致的长期持续一系/多系血细胞减低并未显著增加新冠感染重症发生率。

通过对比IST组与非IST组感染早期的临床特征，我们发现AA患者是否接受IST对于新冠感染的早期临床症状并无明显影响。与IST<6个月患者相比，IST≥6个月的患者在新冠感染早期并未出现更重的发热、呼吸道及消化道症状。而且接受更强清除T细胞的IST并未导致新冠感染早期症状加重。提示AA患者的早期新冠感染症状与是否接受IST、IST强度及时长并无显著相关性。

通过对新冠感染恢复期临床症状分析，我们发现发热、头痛、肌肉酸痛等症状多出现于感染早期，咳嗽/咳痰等呼吸道症状多发于感染恢复期，且集中于感染后1周。是否接受IST对新冠感染后1周、2周的症状无明显影响。IST≥6个月与IST<6个月患者的感染恢复期症状差异无统计学意义。但在接受强IST患者中，新冠感染1周后发热比例更低，感染2周后咽痛比例更高，可能与T细胞清除后早期免疫应答减弱且病毒清除能力减弱有关。

目前国内外仅有部分针对AA合并新冠感染的病例报道[6]–[12]和一项回顾性研究[13]，尚无针对大规模前瞻性队列研究。本研究基于中国第一个前瞻性纵向血液病队列（NICHE队列），详细描述了AA患者不同免疫抑制治疗状态下合并新冠感染的临床特征及转归情况，为AA患者治疗过程中合并流行性疾病的管理提供了高质量循证医学证据。本研究也存在一定局限性，研究主要通过网络平台发放电子问卷进行流行病学调查和随访，部分AA患者如文化程度低、年龄较大且不擅长使用网络的患者，以及一些依从性较差无应答的患者未纳入研究。
